# The importance of clinical importance when determining the target difference in sample size calculations

**DOI:** 10.1186/s13063-023-07532-5

**Published:** 2023-08-04

**Authors:** Richard A. Parker, Jonathan A. Cook

**Affiliations:** 1 Edinburgh Clinical Trials Unit, Usher Institute, University of Edinburgh, Edinburgh, UK; 2 Centre for Statistics in Medicine, Nuffield Department of Orthopaedics, Rheumatology and Musculoskeletal Sciences, University of Oxford, Oxford, UK

**Keywords:** Clinical trial, RCT, Power, Effect size, Assumed benefit, Clinically relevant difference, Minimum important difference, Target difference

## Abstract

Recently, it was argued that clinically important differences should play no role in sample size calculations. Instead, it was proposed that sample size calculations should focus on setting realistic estimates of treatment benefit. We disagree, and argue in this article that considering the importance of a target difference is necessary in the context of randomised controlled trials of effectiveness, particularly definitive phase III trials. Ignoring clinical importance could have serious ethical and practical consequences.

## Introduction

We read with interest the article by Wong regarding specification of the target difference in sample size calculations [1]. Wong suggests that the focus of sample size estimation should be solely on a “realistic estimate of benefit”. Furthermore, he suggests that the validity of the calculation can usefully be understood in terms of “true power” [1]. We disagree with both assertions, and outline our concerns with his proposal below. To begin with, we note our focus in this article will be primarily framed around the use of the conventional (Neyman-Pearson) approach to determining the sample size for a randomised controlled trial (RCT), although our rationale and concerns outlined below also apply more broadly (e.g. to sample size calculations within a Bayesian framework) [2]. The focus here will be on what might be considered phase III, or definitive, RCTs. Finally, we define the target difference as the difference in the outcome of interest (e.g. primary outcome) we would like to be able to detect with reasonable certainty in the RCT analysis. We note that selecting an appropriate target difference value is vital as calculated sample sizes are very sensitive to its magnitude [3, 4]. For example, halving the target difference quadruples the required sample size for a standard (2-arm parallel group) RCT using a standard calculation for a continuous outcome [5].

## Are realistic differences enough?

Wong posits that when constructing sample size calculations, investigators should only be focussed on determining realistic estimates of the target difference and that the “minimum important difference should play no role in setting the sample size” [1]. As noted by Wong, and which we readily acknowledge, determining the clinical importance of a difference in an outcome, particularly the *minimum* clinical importance, is far from straightforward [6–12]. However, we disagree with the position that clinical importance is of no value in determining the sample size for a RCT. We argue instead that the clinical importance of the target difference in a sample size calculation (defined according to a stakeholder group of interest) deserves prominent consideration, particularly in the context of phase III, or definitive, RCTs. The basis for this is discussed with reference to the purpose of sample size calculations, as outlined below.

## The rationale for sample size calculations

Wong argues that “setting the assumed benefit to the MID [minimum important difference] clearly is inadequate for generating strong evidence for a MID benefit” [1]. However, the rationale for most trials is *not* that we determine conclusively that the true treatment difference is greater than the clinically relevance threshold; but that *if* the true difference is clinically relevant, then we have sufficient sample size to reach a conclusion of statistical significance and estimate the treatment effect with adequate precision. There is nothing wrong with basing a sample size calculation on a hypothetical target difference (assumed benefit) without any knowledge as to whether it really exists. Indeed, this is *why* we are conducting the trial in the first place. The point is that *if* a hypothetical true difference exists, and it is minimal clinically relevant, there is sufficient power to detect it.

A related point is that it is often very difficult to determine what is, and what is not, a “realistic” difference. Little or no prior work may have been conducted to guide the investigator, or even if prior work has been done, treatment effects may not be reliable because they are based on small sample sizes, publication bias, and/or within-study bias. The lack of certainty is the reason for conducting the trial. We agree with Wong that a range of values for the target difference should be considered when designing sample size calculations [1], but we would argue that the importance of the potential values should be a factor in establishing the sample size that is ultimately chosen. Certainly, in our view, this should be the case for any RCT that is conducted with a view to influencing clinical practice or related policy-making.

## Sample size validity

Wong discusses sample size “validity” and asserts that a sample size is valid when the “value for the assumed benefit is close to the true benefit” [1]. This implies that the validity of the trial increases when the assumed benefit is closest to the true benefit. However, this statement does not make sense when taken to its logical conclusion. The assumed benefit is closest to the true benefit when our knowledge about the true benefit increases. However, if we knew the true benefit precisely, then there is no point in doing the trial! Indeed, as our knowledge of the true benefit increases, the *less* relevant a sample size calculation becomes. For this reason, the arguments made with reference to a true benefit “near 4 units” (as per Wong’s hypothetical example [1]) are not cogent, because no one ever knows the true benefit in practice; or if they did, there would be no point in doing the trial. More generally, we reject this conception of the “validity” of a sample size calculation. The analysis, if conducted appropriately, will be valid irrespective of the sample size. If we pin the sample size exclusively on a “realistic” basis, then we may guess the target difference well or poorly, but it is wrong in our view to conceptualise this as being “valid” or not. It may lead to a study which does not progress our understanding, but that is not an issue of statistical or scientific validity but one of value. Ultimately, the value of an individual study can only be understood well after its conduct and in the context of other evidence and how science and practice may change.

Consider a trial where the true benefit is zero, and we are testing against a null hypothesis of zero difference between the treatment groups. In this case, the required sample size tends to infinity the closer the assumed benefit gets to the true benefit. To be “valid” according to the criterion proposed by Wong, we need to have a sample size which is based upon a very small magnitude. If a numerically small value is used, this will lead to an impossibly large trial. For example, a target mean difference of 0.001 (with a standard deviation of 1, 80% statistical power, and 2-sided 5 significance level) requires over 3 million observations! If we were to relent and use a loose requirement of “nearly” zero, we are back in the ballpark of defining what is “near” enough which depends upon the scale. A difference of 1.0 in the EQ-5D 5L index (https://euroqol.org/) is very different than a 1.0 difference in diastolic blood pressure (measured in mm Hg). This highlights the ultimate futility of seeking to ensure the assumed benefit is close to the true benefit as this may well be zero (or very close to zero).

The goal of a conventional sample size calculation is not to have the “target power” that “matches” the “true power”, but rather to ensure that if an appropriate hypothetical target difference exists, we are likely to detect it. In the conventional (Neyman-Pearson based) sample size calculation, this is framed in terms of statistical power, though the same approach can be reframed for designs where a different approach is taken. For example, a Bayesian design might be adopted, and the analysis may seek to estimate the Bayesian probability of an effect in favour of one treatment [2]. In this case, the sample size is still driven by the assumed posterior distribution of the true benefit. Although we agree that a “realistic difference” is a key consideration—after all, there is no point in targeting a difference that is completely unrealistic—we disagree that it is by default the sole or the most critical consideration. Indeed, we would argue that investigators should not strive to detect differences that are “realistic” at the expense of clinical importance when seeking to inform clinical practice and policy. In contrast to Wong, we do believe that there is a role in attempting to “reconcile discrepancies in judgments between what is important and what is realistic” [1] at the outset when designing a definitive RCT, provided that a target difference still remains clinically important. We agree with Fayers et al. [13] that the target difference should usually be specified to be both important and realistic [13, 14]. As the DELTA-2 guidance states, “the target difference for a definitive trial (e.g. phase III) should be one considered to be important to at least one key stakeholder group” [3]. Stakeholder groups include patients, health professionals, regulatory agencies, and healthcare funders [3]. Relevant literature could also play a role (if available) in informing what is both an important and realistic difference [3]. Besides the value being important and realistic, the target difference should also be chosen to be consistent with the population-level summary of a pre-defined estimand and reflect the planned analysis [5, 15]. At least one stakeholder group may be involved in defining an appropriate estimand [16], and this also may provide an opportunity to ask them about the importance of the target difference. Involving stakeholder groups when defining both the estimand and target difference will help ensure that research and statistical analyses are both impactful and useful to stakeholders, whereas ignoring the interests of stakeholder groups may lead to research that is ethically dubious or at least not useful.

## Ethical issues

The sample size calculation has another important implicit role regarding upholding appropriate ethical and scientific standards. The purpose of a sample size calculation is not only to ensure that the trial will “generate sufficient information” [1] to inform clinical practice, but also to ensure that the sample size is not too large that it constitutes a waste of resources or leads to unnecessary patient burden [4, 17, 18]. There is also a need to avoid exposing excess patients to the uncertainty and research risks inherent in any trial [4, 5, 17]. Of course, some trials will expose patients to more risk and/or burden than others, and so for these trials, the need to minimise the sample size is more acute. There are also trials recruiting patients with very serious conditions (e.g. motor neuron disease or cancer), often resulting in significant morbidity or mortality within a short time frame. For trials operating in these areas in particular, there is a great need to get answers as quickly as possible. Basing a sample size purely on what is deemed to be the most realistic target difference may be scientifically interesting, but we would argue it is not in the best interest of the patient if it leads to the design of trials that are larger and costlier than necessary, and subsequently take longer to complete. After all, how can a trial based around a small effect size be justified if the target difference is not meaningful to patients, health professionals, or other stakeholders?

Take for example the MND-SMART adaptive design trial [19, 20], a phase III trial currently investigating new treatments for patients suffering from motor neuron disease (MND). Co-primary outcomes are a measure of MND-related disability (the Amyotrophic Lateral Sclerosis Functional Rating Scale Revised (ALS-FRS-R)) [21] and survival. In this context, only clinically relevant treatment effects are of interest (e.g. improved function and mortality). There is no need to precisely measure the treatment effect on ALS-FRS-R in this trial if it is “realistic” but does not have any tangible effect on disability or survival in these patients. Therefore, it makes no sense to determine the sample size characteristics of the MND-SMART trial without reference to clinical importance and only focusing on what is deemed to be realistic. *If and only if* the potential treatment effects are clinically important are we interested in ensuring we have sufficient sample size to make robust conclusions about clinical effectiveness. Conducting a very large trial to detect a difference that falls below the margin of clinical relevance could be potentially unethical from the perspectives of patients and clinicians involved in medical research studies. At the very least, it would be wasteful of precious resources and patients’ support. In this context, the overriding concern is searching for novel treatments that will improve patients’ lives and make an impact on clinical practice. Target differences for phase III trials need to be clinically important in order to minimise the number of patients needlessly taking part in the trial, as well as allowing us to reach a conclusion regarding treatment effectiveness as quickly as possible. As has been stated elsewhere by Cook et al., it is “an ethically imperative that an appropriate number of study participants be recruited, to avoid imposing the burdens of a clinical trial on more patients than necessary” [5].

Suppose that the true benefit of the treatment is not sufficient to be clinically important but exactly equals the “realistic” difference that was chosen as the target difference in our sample size calculation. According to Wong’s terminology, this sample size would be deemed “valid”. However, our sample size is larger than necessary and will identify a statistically significant result on the basis of differences that are not clinically relevant, potentially not even close. Besides the obvious ethical issues, this makes the interpretation of the trial more difficult: statistical significance could be generated on the basis of irrelevant differences.

The ethical impact of sample size overestimation is that the involvement of at least some of the trial participants would be needless, wasteful of their time, and potentially exposing them to unnecessary risks. Trial participants are often inclined to take part in a clinical trial if they feel they are making a difference to other patients diagnosed with the condition in future and/or they are helping to promote advancement in medical knowledge [22]. In this case, their involvement in the trial would arguably not meet  their hopes or expectations. Indeed, avoiding overestimation of sample size is important if we want to avoid the scandal of bad research [23]. Moreover, from a purely practical point of view, it is worth noting that many trials fail to reach their target recruitment and therefore an unnecessarily large sample size target is not appealing.

## The cost of a trial

Setting up and delivering a clinical trial is expensive not only in terms of monetary cost, but also in terms of the necessary input of time and resources. The outputs and impact of a clinical trial have to be worth the substantial investment involved. For this reason, there is a downward pressure on the clinically relevant target difference. Ensuring the trial is likely to add meaningfully to the existing literature and provides a useful result in its own right, while being deliverable in terms of the cost mentioned above can be a delicate balancing act. Some trials may not be undertaken because they are too expensive or too difficult to deliver based on detecting the smallest clinically relevant differences. For example, in the context of common diseases such as stroke, very small reductions in mortality may still be clinically relevant when considering the large population that the treatment will be applied to, and yet this might potentially lead to a trial of a prohibitive size.

We also have to consider that although on the theoretical or scientific level a trial may be well-powered at the study design stage, in practice, the sample size assumptions may not be met such that the trial analysis of the primary outcome may lack precision or have inconclusive findings on its completion. It is advisable to use conservative values for the sample size parameters used (e.g. assumed standard deviation), to ensure the trial results are sufficiently informative and confidence intervals sufficiently narrow in the event that the sample size assumptions are slightly different than planned. However, this luxury is often not practical given the potential impact upon the sample size and corresponding cost to deliver. Nevertheless, it is often useful to test the sensitivity of sample size calculations with respect to their assumptions [14, 24]. Sample size re-estimation is also an option, as suggested by Wong [1].

## Conclusions

In summary, we argue that considering the importance of a target difference is crucial when constructing a sample size calculation in the context of RCTs of effectiveness (especially in phase III trials). Indeed, ignoring clinical importance could have serious ethical and practical consequences because the resulting sample size may be larger than necessary. The true treatment effect in medical research is the very thing which the trial is intended to estimate, and which stakeholders are interested in. It is very difficult to assess how closely an assumed target difference agrees with the true difference, and even if it were possible to do this, we encounter the irrationality of trying to construct a sample size calculation based on a true difference that we already know about. In our opinion, researchers should be more concerned with determining the sample size upon a credible basis, and analysing and reporting what they have done in an appropriate and informative manner, than worrying about whether their target difference is close to the true difference.

## Data Availability

Not applicable.
